# Implementation of a Load Sensitizing Bridge Spherical Bearing Based on Low-Coherent Fiber-Optic Sensors Combined with Neural Network Algorithms

**DOI:** 10.3390/s21010037

**Published:** 2020-12-23

**Authors:** Jingjing Guo, Tiesuo Geng, Huaizhi Yan, Lize Du, Zhe Zhang, Changsen Sun

**Affiliations:** 1 School of Optoelectronic Engineering and Instrumentation Science, Dalian University of Technology, Dalian 116024, China; guocongcong@mail.dlut.edu.cn (J.G.); yhzmyymyhz@163.com (H.Y.); dulize528@163.com (L.D.); 2 Bridge and Tunnel Research and Development Center, Dalian University of Technology, Dalian 116024, China; gengts@dlut.edu.cn (T.G.); zhangzhe@dlut.edu.cn (Z.Z.)

**Keywords:** bridge bearing, fiber-optic sensor, load-sensitizing, neutral network algorithm

## Abstract

Low-coherent fiber-optic sensors combined with neural network algorithms were designed to carry out a load-sensitizing spherical bearing. Four sensing fibers were wound around the outside of the pot support of the spherical bearing uniformly deployed from upper to bottom. The upper three were configured in a distributed way to respond to the applied load as a function of the three strain sensors. The bottom one was employed as a temperature compensation sensor. A loading experiment was implemented to test the performance of the designed system. The results showed that there was a hysteresis in all the three sensors between loading and unloading process. The neural network algorithm is proposed to set up a function of the three sensors, treated as a set of input vectors to establish the input-output relationship between the applied loads and the constructed input vectors, in order to overcome the hysteresis existing in each sensor. An accuracy of 6% for load sensing was approached after temperature compensation.

## 1. Introduction

Structural health monitoring (SHM) has become a significant approach to ensure the safety of in-service civil structures, especially for railway bridges [1]. As an important element, bridge bearings can ensure the load transmission from deck to support and provide a certain degree of freedom for sliding and rotating. The bridge bearings reflect the static, dynamic and even the seismic performance of the bridge, and to a great extent determine the lifetime of the bridge. Therefore, it is important to develop a technique to monitor the loading status in real time by monitoring the bridge bearing.

The piezoelectric method was employed to monitor the bridge bearing [2,3]. The advantage was its passive properties; the drawback was its suffering from electromagnetic interference. Caussignac et al. [4,5] introduced a fiber-optic sensor technique to overcome interference in order to implement measurement in harsher engineering situations. Based on these studies, Y. Zhuang et al. related the performance of the bearing to the structural response of the bridge by finite element simulation [6]. All the studies introduced above are based on elastomer bearing under lower load conditions, because of its simple structure and fairly linear mechanical property.

With increase of bridge load level, the application of spherical steel bearings has become more and more widespread, because that spherical bearings can accommodate larger vertical loads, displacements and rotations [7], and are more resistant to low temperatures. Thus, it is necessary and meaningful to monitor the spherical steel bearing underlying a large load. However, hysteresis loops [8,9,10] have become an inevitable obstacle to design a load-sensitizing bridge spherical bearing, even if it facilitates vibration isolation and energy dissipation [11]. Therefore, studies on large-load sensitizing spherical bearing are still making limited progress. 

In this paper, we propose a low-coherent fiber-optic sensor (LCFS) [12,13,14] combined with neural network (NN) algorithms to carry out a load-sensitizing spherical bearing. The sensor was composed of three optical fibers, which responded to the applied loading simultaneously but with different strain responsive factors. The factors were determined by the wound position of the three sensing fibers around the outside of the pot support of the spherical bearing from upper to bottom. The strain sensitivity of LCFS can be improved by increasing the sensing fiber length, which makes it easier to achieve high resolution and realize early detection of the structure. A neural network algorithm was designed to compensate for the hysteresis.

The NN technique in the field of SHM has been employed to predict damage in a complex structure, bridge deflection and structural crack location, etc. [15,16,17]. With a remarkable ability to derive meanings from complicated or imprecise data, the NN technique makes it possible to solve problems in patterns extraction and trend detection which are too complex to be treated by theoretical analysis. The Back-Propagation Neural Network (BPNN) has been applied successfully to solve difficult and diverse problems by training the network in a supervised manner with the error back-propagation algorithm [18]. Here, the three strain sensors were configured as a function of the applied loading and to act as an input of the NN analysis program. The experimental tests demonstrated that an accuracy of 6% for load measurement was approached after temperature compensation. 

## 2. Theoretical Background

### 2.1. Strain Sensors Based on Scanning Fiber-Optic Low-Coherent Michelson Interferometer (SLMI)

**Figure 1:**
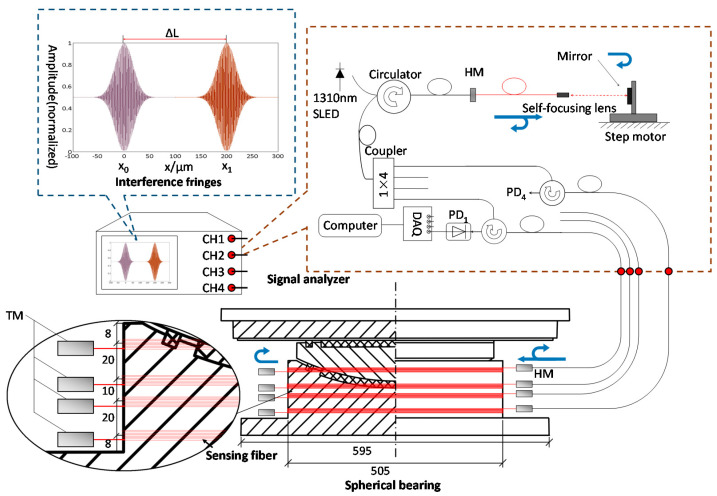
The diagram of the SLMI measurement system connecting with the spherical bearing. SLED: super-luminescent emitting diode; HM: half-reflection mirrors; TM: total mirrors; PD: photonic-detector; DAQ: data acquisition system.

The proposed sensors in this work are mainly based on the use of SLMI [13,14]. As a type of long-gauge fiber-optic sensor, it is typically used for corrosion [12], strain/deformation [19] and settlement [14,20] measurements of large structures. The structure and size of the spherical bearing and the configuration of the sensing system are shown in Figure 1. Four fiber-optic sensors for load sensing are wound around the outside of the pot support of the spherical bearing (QKQZ-I-5000) alternatively from upper to lower. The upper three will present a decreasing strain factor as the location becomes lower. The fourth sensor was designed to show little response to the loading and acted as a temperature compensation.

The SLMI works with a light beam emitted by a super-luminescent emitting diode (SLED) source with a central wavelength of 1310 nm and a bandwidth of 50 nm, transmitted through several circulators, fiber-optic half-reflection mirrors (HM) and total mirrors (TM), which form a fiber-optic Michelson interferometer. The interference signal is detected by a photonic detector (PD) and then acquired by a data acquisition system (DAQ).

As discussed in [12], the initial length of the optical fiber will be recorded as *x*_0_ by the interferometer demodulation. When a load is applied to the spherical bearing, the strain applied to the sensing fiber changes, and the changed optical fiber length, will be recorded as *x*_1_. The deformation can be described as:

(1) $$\Delta L=\frac{\left|x_{1}-x_{0}\right|}{n}$$

where, *n* is the refractive index of the sensing fiber.

The average strain of the spherical bearing side, *ε*, can be calculated as:

(2) $$\varepsilon =\frac{\Delta L}{L_{s}}$$

where, *L*_s_ is the length of sensing fiber. In this study, the 7.93 m-length sensing fiber was wound around the outside pot support of the spherical bearing, which was of 505 mm diameter, and about five turns of fiber formed each sensor.

### 2.2. The Back-Propagation Neural Network

**Figure 2:**
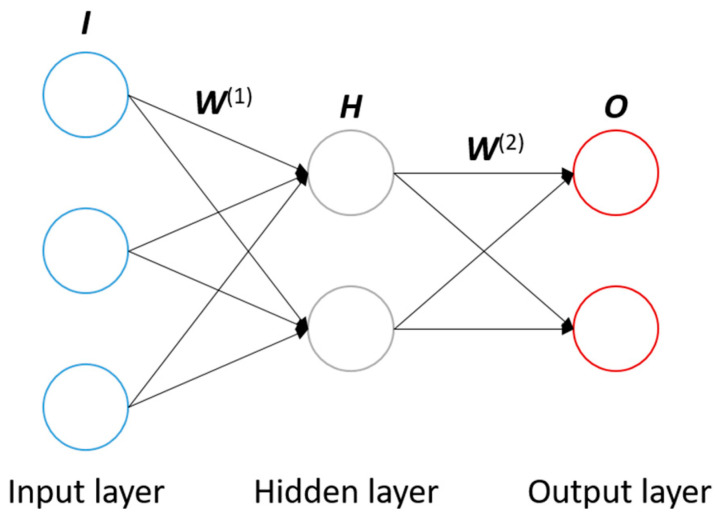
Diagram of the NN topography.

The NN technique is an important method for pattern recognition, classification, and function approximation, and has attracted much attention in the field [21,22,23,24]. In this study, the NN technique was employed to establish the relationship between the strain and the load of spherical bearing. The basic principles and construction process are briefly introduced below (Figure 2).

A classic 3-layer NN topology is shown in Figure 2. The input layer, hidden layer and output layer comprise the neuron network. Each node in the diagram represents a neuron. The number of nodes in input layer and output layer is determined by characteristics of data, while the number of nodes in the hidden layer could set according to the requirements. The function of the neural network is to predict unknown attributes via the known attributes of the samples. In Figure 2, there are three attributes as nodes in the input layer, and two unknown attributes as nodes in the output layer. The connections between nodes represent the weights, and all the connections between layers compose the weight matrices, ***W***^(1)^ and ***W***^(2)^, which determine the calculation process between layers. Suppose there are 20 samples, each of the known attributes can form the vector ***i*** with 20 elements, and three known attributes can be written as ***i***_1_, ***i***_2_ and ***i***_3_. Therefore, the input layer can be written as a matrix ***I*** as follows:

(3) $$I=\left[\begin{array}{c} i_{1}\\ i_{2}\\ i_{3} \end{array}\right]=\left[\begin{array}{ccc} i_{1,1} & \cdots & i_{1,20}\\ i_{2,1} & \cdots & i_{2,20}\\ i_{3,1} & \cdots & i_{3,20} \end{array}\right]$$

The hidden layer matrix ***H*** is calculated by:

(4) $$H=g(W^{\left(1\right)}•I)=g\left(\left[\begin{array}{ccc} w_{1,1}^{\left(1\right)} & w_{1,2}^{\left(1\right)} & w_{1,3}^{\left(1\right)}\\ \vdots & \vdots & \vdots \\ w_{15,1}^{\left(1\right)} & w_{15,2}^{\left(1\right)} & w_{15,3}^{\left(1\right)} \end{array}\right]\left[\begin{array}{ccc} i_{1,1} & \cdots & i_{1,20}\\ i_{2,1} & \cdots & i_{2,20}\\ i_{3,1} & \cdots & i_{3,20} \end{array}\right]\right)=\left[\begin{array}{ccc} h_{1,1} & \ldots & h_{1,20}\\ \vdots & \ddots & \vdots \\ h_{15,1} & \cdots & h_{15,20} \end{array}\right]$$

where *g* is the sigmoid function, defined by:

(5) $$g\left(x\right)=\frac{1}{1+e^{-x}}$$

Similarly, the output layer consists of two elements, which can be written as matrix ***O***:

(6) $$O=\left[\begin{array}{c} o_{1}\\ o_{2} \end{array}\right]=\left[\begin{array}{ccc} o_{1,1} & \cdots & o_{1,20}\\ o_{2,1} & \cdots & o_{2,20} \end{array}\right]$$

The output layer matrix ***O*** is calculated by:

(7) $$O=g(W^{\left(2\right)}•H)=g\left(\left[\begin{array}{ccc} w_{1,1}^{\left(2\right)} & \cdots & w_{1,15}^{\left(2\right)}\\ w_{2,1}^{\left(2\right)} & \cdots & w_{2,15}^{\left(2\right)} \end{array}\right]\left[\begin{array}{ccc} h_{1,1} & \ldots & h_{1,20}\\ \vdots & \ddots & \vdots \\ h_{15,1} & \cdots & h_{15,20} \end{array}\right]\right)$$

Then the weight matrices are modified to ensure that the predicted results are sufficiently close to the desired results by the training process. The total error in the performance of the network, ***E***, is defined as:

(8) $$E=\frac{1}{2}{\left\|O-O_{p}\right\|}^{2}$$

where ***O****_p_* denotes the desired output results matrix. In order to minimize ***E*** by gradient descent, it is necessary to compute the partial derivative of ***E*** for each weight in the network. The structure of the neural network is usually complicated, so the direct calculation of partial derivation is also computationally intensive. To overcome the limitation, a BPNN algorithm [25] based on chain rule is employed in NN training. The BPNN algorithm utilizes the structure of the network to calculate the partial derivative from back to forward. Firstly, the gradient between output layer and hidden layer is calculated to modify the weight matrix ***W***^(2)^. Then the gradient between hidden layer and input layer is calculated to modify the weight matrix ***W***^(1)^. The modification process occurs iteratively until the accuracy requirements are reached, and then the trained neural network is employed to predict the unknown attributes of the samples.

## 3. Experimental Study

### 3.1. Finite Element Analysis

**Figure 3:**
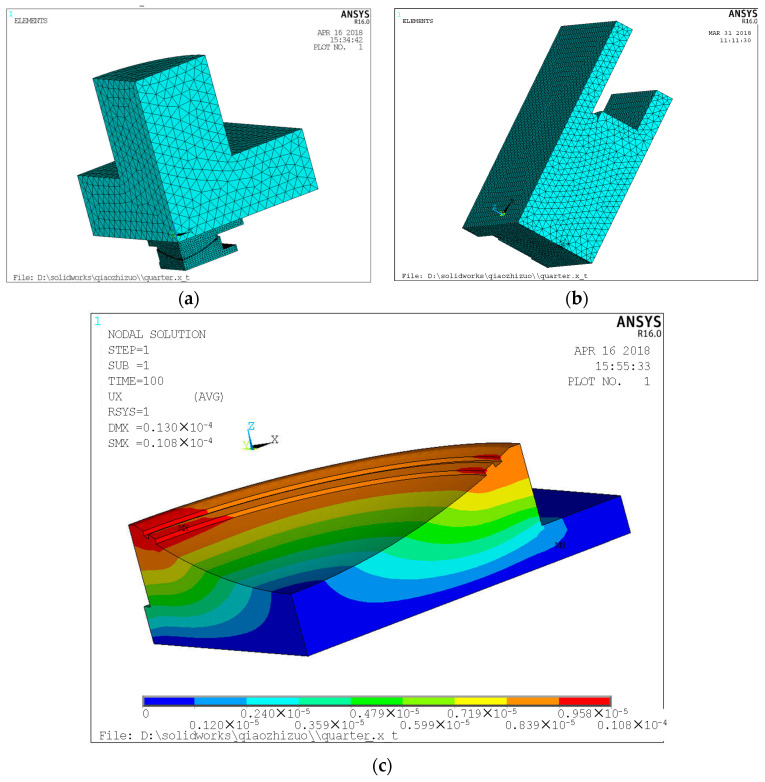
The finite element model and the analysis results. (**a**) The finite element model grid mesh division. (**b**) The grid mesh model of spherical bearing. (**c**) The finite element model analysis results.

The finite element analysis (FEA) method has been widely used to simulate the wear processes of mechanical parts [26]. For an optimal sensor installation, a 3D FEA model of the spherical bearing was developed using ANSYS to simulate the static deformation and strain of the spherical bearing under a load of 5000 kN, as shown in Figure 3a. The upper section of the model is the schematic of the test machine applying the load. The lower section of the model is the schematic of the spherical bearing as shown in Figure 3b. The parameters of this model are set as shown in Table 1.

The simulation results of the spherical bearing at 5000 kN are shown in Figure 3c. Based on the results, location-dependent and uneven strain features was observed. The maximum of the bearing pot strain is located on the upper side of the bearing pot support, and the minimum is located on the bottom side of the bearing. Local tensile stress occurs between the upper support slate and the spherical slider when the force transit is from upper to lower, and the same process occurs between the spherical slider and the lower bearing slate, which results in deformation of the spherical bearing. 

**Figure 4:**
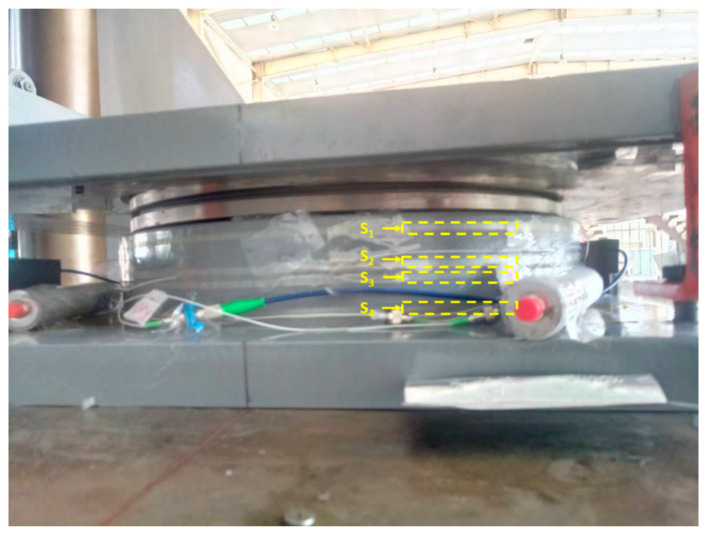
The picture view of load-sensitizing spherical bearing. S_1,_ S_2,_ S_3_: the three sensing fibers located at the upper region of the pot support; S_4_: the reference fiber, acting as a temperature compensation fiber.

Consequently, the reference fiber should be designed as close to the bottom side of the bearing pot support as possible to form an approximately load-independent temperature compensation. Meanwhile, the other three sensing optical fibers were wound at different positions of the pot support to measure comprehensive deformation of the spherical bearing. As shown in Figure 4, S_1_, S_2_ and S_3_ were chosen as sensing fibers, and S_4_ was chosen as reference fiber.

### 3.2. Loading Experiments

**Figure 5:**
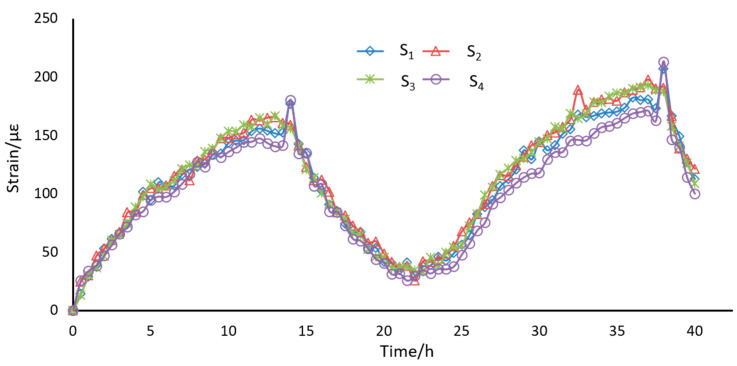
The long-term stability of the designed sensors.

**Figure 6:**
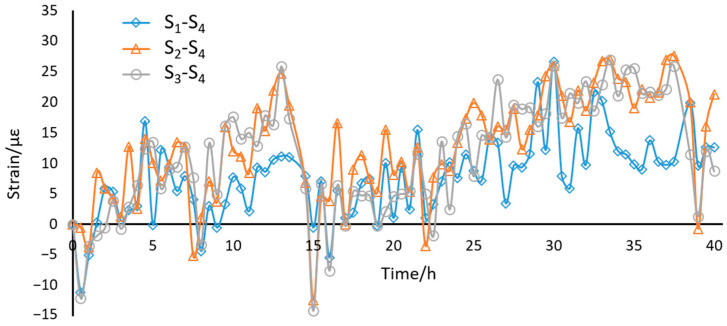
The results after being compensated by S_4_.

We tested the long-term stability of the sensors by putting in an open room for 48 h without applying any loading. As shown in Figure 5, the results of four sensors varied from 0 to 250 με with the room temperature fluctuations. In order to check the compensation performance of the reference sensor, a subtraction was implemented between measuring sensors and reference sensor. The results were presented in Figure 6, in which a fluctuation from −15 με to 30 με was recorded. The temperature effects were greatly suppressed by the compensation sensor.

**Figure 7:**
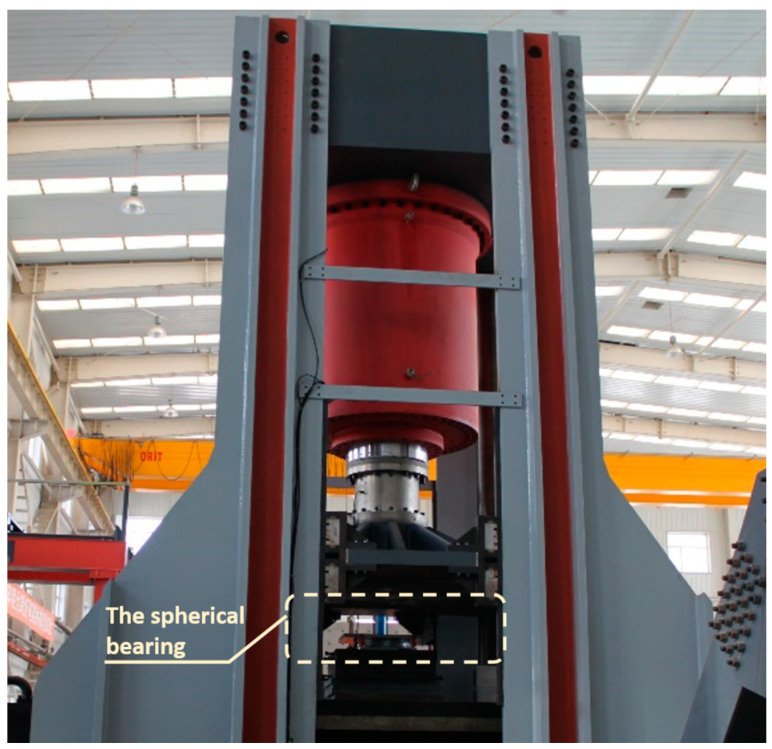
YAJ-30000 KN microcomputer controlled electro-hydraulic servo press shear test machine.

**Figure 8:**
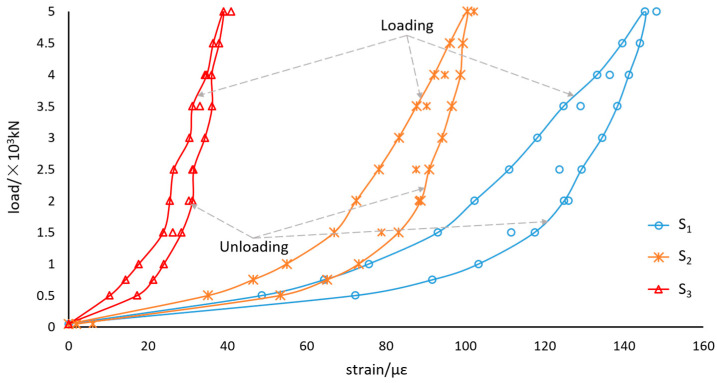
Fiber-optic sensors response of the bearing under the loading and unloading.

In order to experimentally determine the correspondence in behavior between the fiber-optic sensors and bearing, the loading experiment was implemented on a YAJ-30000 kN test machine as shown in Figure 7. Figure 8 shows the strain-load diagram, which is due to a loading circle by increments. The load ramped from 50 kN to 5000 kN by steps of 500 kN and was then restored to 50 kN by the same step. Random loads were applied to the bearing at 50 kN, 4000 kN, 3500 kN, 1500 kN, 5000 kN, 2500 kN and 50 kN. Each point corresponds to an instantaneous value, which is obtained with fiber-optic sensors 2 min after the load is constant.

## 4. Discussion

Figure 8 describes some aspects of the fiber-optic sensors’ response to the loading and unloading: the fiber-optic sensors and bearing deformations agree in behavior; the sensor S_1_ gives the greatest response of 145.25 με under the load of 5000 kN, which seems to be the most suitable sensor for the bearing load measurement; the loading curves and unloading curves are inconsistent, and form hysteresis loops, which is not conductive to the loading calculation. 

**Figure 9:**
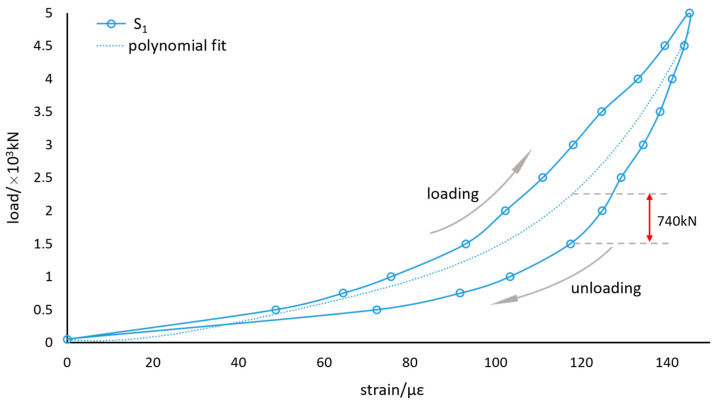
Polynomial fitting of the load-strain relationship.

The load-strain diagram of the sensor S_1_ is shown in Figure 9. The polynomial fitting curves are calculated by the programs to predict the load applied to the bearings. Because of the non-coincidence between the loading curve and the unloading curve, the deviation between the measured value and the calculated value is as high as 740 kN, which is far from the required precision.

It is insufficient to calculate the bearing loads by any single sensor. However, as shown in Figure 8, the three loading curves are always below the unloading curves. To some extent, the relationship between the three sensors changes synchronously with the load. Therefore, we believe that if the three sensors are regarded as a ternary vector, there may be a unique mapping between the vectors and the loads applied to the bearing, which are only related to loading values other than in the process. We can establish an implicit function mapping between strains and loads. The implicit function can be described as:

(9) $$F=f\left(s_{1},s_{2},s_{3}\right)$$

where *F* is the load applied to the spherical bearing, and *s_1_*, *s*_2_, *s*_3_ are the strain data obtained by the fiber-optic sensors.

**Figure 10:**
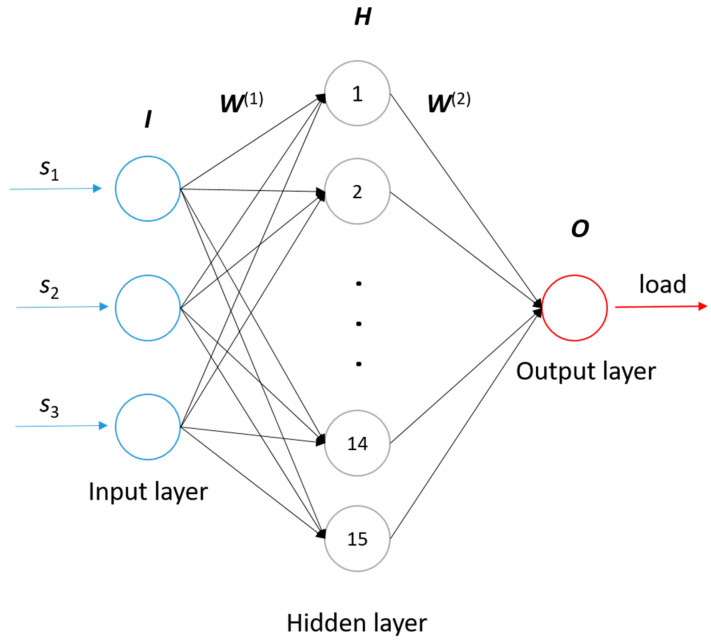
The employed back-propagation neural network (BPNN) model to predict loads.

In this study, a BPNN algorithm is employed to establish the relationship *f* between strains and loads. We built a 3-layer BPNN model, as shown in Figure 10. The number of nodes is set to 15. The sigmoid function is selected as the transfer function *g*. In the loading circle experiment by increments, 23 loads were applied to the spherical bearing. Therefore, the strain data measured by 3 fiber-optic sensors, S_1_, S_2_ and S_3_, under the loading circle formed an input matrix ***I*** of 3 × 23 size, and the loads applied to the spherical bearing formed an output matrix ***O*** of 1 × 23 size. These two matrices are set as input layer and output layer of the NN, respectively. As described as Section 2.2, the calculation process from the input layer to the output layer is:

(10) $$H_{15\times 23}=g(W_{15\times 3}^{\left(1\right)}•I_{3\times 23})$$

(11) $$O_{1\times 23}=g(W_{1\times 15}^{\left(2\right)}•H_{15\times 23})$$

where the size of weight matrices ***W***^(1)^, ***W***^(2)^ are determined by nodes of the adjacent layers.

**Figure 11:**
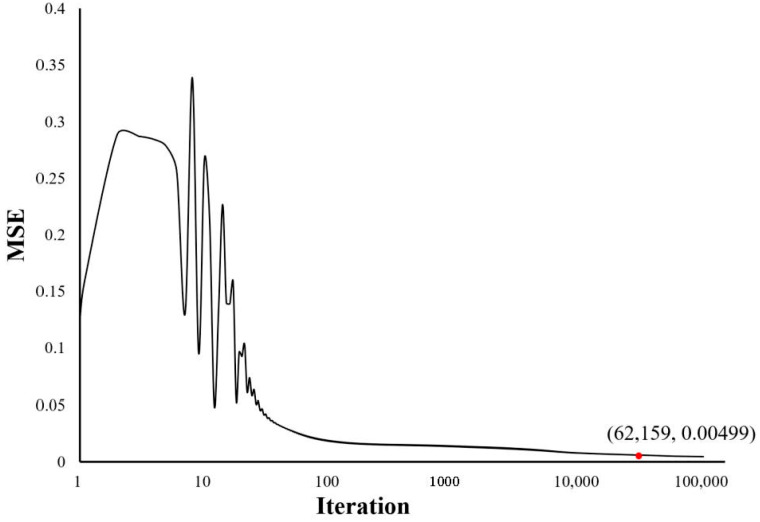
Influence of iteration number on the performance of BPNN.

The strain data measured in the incremental loading cycle experiment were selected as the training dataset to train the NN. Here, mean square error (MSE) is employed to judge whether the training has been completed. Figure 11 demonstrates the effect of iteration number on MSE. In order to predict the load as accurately as possible, we take a small value, 0.005, as the threshold of MSE, and the corresponding iteration number is 62,159. Thus, the NN training was regarded as completed and stopped here. Then, the strain data measured under the random loads were selected as the testing dataset to confirm the prediction accuracy of the NN. Table 2 shows the loads predicted by the trained NN and the deviations between the predicted results and the actual results.

This shows that the maximum predicted deviations in the NN model is 260 kN, which is far less than the deviations by polynomial fitting. Based on the BPNN algorithm, the precision of the designed intelligential load-sensitizing spherical bearing can meet the measurement requirements. In order to pursue higher precision of the intelligential load-sensitizing spherical bearing, further work will be conducted in the future to improve the accuracy of strain measurements and optimize the BPNN algorithm.

## 5. Conclusions

In this study, we designed an intelligential load-sensitizing spherical bearing based on an optical interferometer, which was considered feasible for monitoring the applied loads. The proposed intelligential bearing was tested in a 30,000 kN press-shear test machine to obtain a set of load-strain data. Based on the experimental dataset, an NN model was trained to predict the loads imposed on the bearing. An accuracy of 6% for load sensing was approached.

Compared to alternative methodologies, the potential drawback of the large load measurement caused by the material response non-coincidence between loading and unloading can be efficiently compensated by the BPNN algorithm. Besides, the main advantage of the proposed intelligential bearing is its simple mechanical preparation method. In other words, the existing structure of the spherical bearing does not need to be changed, and the modification work is only the winding of the fiber-optic sensors around the outside of the bearing pot support. Based on this work, we will conduct practical site experiments in the future to test the performance of the proposed intelligential bearing. Since the complex field environment will cause great interference to the measurement, it is necessary to improve the robustness and flexibility of the NN by adjusting the parameters and NN types and enlarging the datasets. Other machine learning methods are also worthy of comparative and combinative study to enhance the performance of the algorithm. In addition, the proposed intelligential bearing can be further extended to applications of distributed measurement with remote control technology in order to perform huge and complex joint load analysis, and we aim to deduce more comprehensive information, such as that concerning settlement and inclination of the bridge, based on the loads, to assess structural health state.

## Figures and Tables

**Table 1:** The parameters of the designed finite element model.

**Elastic Modulus**		2 × 10^11^ Pa
**Poisson’s Ratio**		0.3
**Friction coefficient**	Upper slate—Lower slate	0.15
	Lower slate—Bridge pier	0.4

**Table 2:** The predicted load by the trained NN and the deviations.

Actual Load/kN	Predicted Load/kN	Deviations
3500	3625	2.5%
1500	1668	3.36%
4000	3783	4.34%
2500	2760	5.20%
5000	4803	3.94%
50	5	0.09%

## Data Availability

Data sharing not applicable.
